# Sunflower Leaf Structure Affects Chlorophyll *a* Fluorescence Induction Kinetics In Vivo

**DOI:** 10.3390/ijms232314996

**Published:** 2022-11-30

**Authors:** Qing-Qing Zou, Dong-Huan Liu, Min Sang, Chuang-Dao Jiang

**Affiliations:** 1 Key Laboratory of Plant Resources, Institute of Botany, Chinese Academy of Sciences, Beijing 100093, China; 2 China National Botanical Garden, Beijing 100093, China; 3 University of Chinese Academy of Sciences, Beijing 100049, China; 4 Beijing Botanical Garden, Beijing Floriculture Engineering Technology Research Centre, Beijing 100093, China

**Keywords:** leaf structure, palisade tissue, spongy tissue, chlorophyll *a* fluorescence induction kinetics

## Abstract

Chlorophyll *a* fluorescence induction kinetics (CFI) is an important tool that reflects the photosynthetic function of leaves, but it remains unclear whether it is affected by leaf structure. Therefore, in this study, the leaf structure and CFI curves of sunflower and sorghum seedlings were analyzed. Results revealed that there was a significant difference between the structures of palisade and spongy tissues in sunflower leaves. Their CFI curves, measured on both the adaxial and abaxial sides, also differed significantly. However, the differences in the leaf structures and CFI curves between both sides of sorghum leaves were not significant. Further analysis revealed that the differences in the CFI curves between the adaxial and abaxial sides of sunflower leaves almost disappeared due to reduced incident light scattering and refraction in the leaf tissues; more importantly, changes in the CFI curves of the abaxial side were greater than the adaxial side. Compared to leaves grown under full sunlight, weak light led to decreased differences in the CFI curves between the adaxial and abaxial sides of sunflower leaves; of these, changes in the CFI curves and palisade tissue structure on the adaxial side were more obvious than on the abaxial side. Therefore, it appears that large differences in sunflower leaf structures may affect the shape of CFI curves. These findings lay a foundation for enhancing our understanding of CFI from a new perspective.

## 1. Introduction

The leaf is the main photosynthetic organ in higher plants. Chlorophyll *a* fluorescence induction kinetics is a highly sensitive, non-destructive, and reliable probe used for studying photosynthesis [1,2]. When dark-adapted leaves are exposed to continuous light, a typical transient of chlorophyll *a* fluorescence can be observed. The time-course of chlorophyll *a* fluorescence is called fast chlorophyll *a* fluorescence induction kinetics (CFI), which reflects the closure of photosystem II (PSII) reaction centers and is related to reductions in Q_A_ to Q_A_^−^. It can thus provide detailed information on the photochemical activity of PSII [1,3,4,5]. Various fluorescence parameters can be calculated based on the shape of the fluorescence-induced kinetic curve and the associated characteristic points (O, I, I, P), which reflect the PSII quantum efficiency and electron transfer activity of leaves [3,4,5]. Under various stress conditions, not only the photosynthetic activity but also the structure (including the shape, size, and arrangement of cells) differ significantly and can vary considerably [6,7,8,9]. Although a few studies have speculated that leaf thickness affects the shape of CFI curves [10,11,12,13], it is unclear whether these structural differences affect the determination and analysis of CFI.

If leaf structure affects CFI, then great differences in leaf structures may result in greater CFI differences. Therefore, the comparison and analysis of leaves or leaf tissues with great structural differences can elucidate this issue. Leaves can be classified into isolateral and bifacial leaves based on their main morphological and structural features. The adaxial and abaxial sides of isolateral leaves have no obvious palisade or spongy tissue differentiation and have similar mesophyll cells; thus, both sides of the leaf can photosynthesize efficiently. Unlike isolateral leaves, the adaxial side of bifacial leaves is composed of tightly arranged columnar palisade cells, while the abaxial side is mainly composed of loosely arranged irregular or globular spongy cells [14,15]. Compared to spongy tissues, palisade tissues are rich in chloroplasts and have a low degree of basal granule stacking. The number of electron transporters in the chloroplasts of palisade cells and the content of enzymes related to carbon assimilation is higher than in spongy cells [16,17,18]. Accordingly, the photochemical efficiency, electron transfer activity, and carbon assimilation activity of palisade cells are higher than spongy tissues, and differences between the palisade and spongy tissue structures and photosynthetic functions are very obvious. Therefore, palisade and spongy tissues can be utilized to analyze the relationship between leaf structure differences and CFI.

In general, light enters leaf tissues from the adaxial side of the leaf. When the incident light on the adaxial side propagates to half the thickness of the palisade tissue inside the leaf, the light intensity decreases by ≥60% [19]. When light completely penetrates the palisade tissues, the incident light intensity may be as low as 10% [19,20,21,22]. Therefore, palisade tissues are more conducive to light incidence and utilization. However, unlike palisade cells, the irregular shape and sparse arrangement of spongy tissue cells easily lead to the refraction and scattering of incident light [23]. Therefore, based on great differences in the photosynthetic function and structure between leaf palisade and spongy tissues, we hypothesized that CFI differences between the adaxial and abaxial sides of bifacial leaves are related to structure, and it is likely that the shapes of the CFI curves are affected by leaf structure on certain extent under environmental stress. In order to investigate our hypothesis, the structure and CFI curves of the adaxial and abaxial sides of leaves in vivo were analyzed to uncover differences in the CFI curves and potential effects of leaf structure on CFI in sunflower (typical bifacial leaf) and sorghum (isolateral leaf) seedlings.

## 2. Results

### 2.1. Differences in the CFI Curves between the Adaxial and Abaxial Sides of Sunflower Leaves

**Figure 1:**
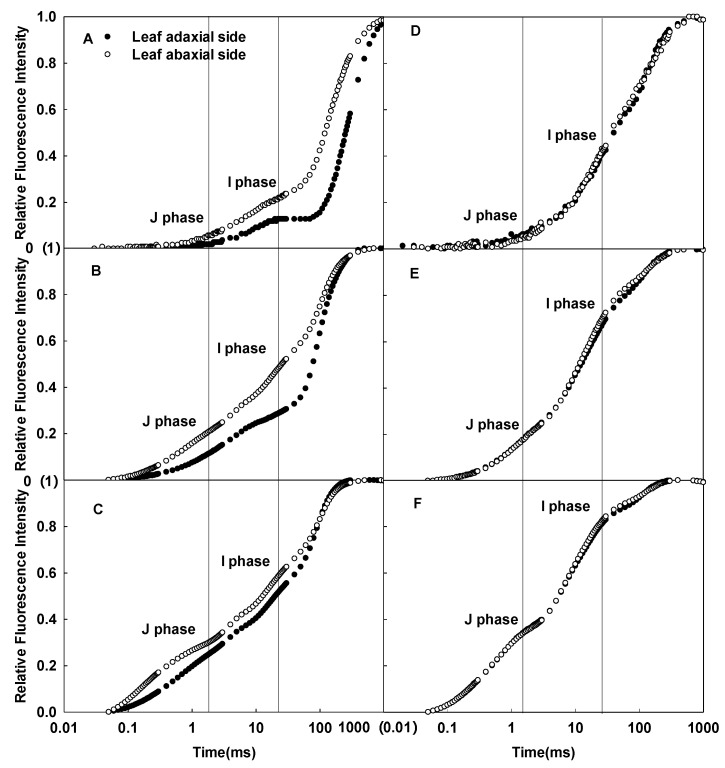
Differences in the CFI curves of the adaxial and abaxial sides of sunflower (**A**–**C**) and sorghum (**D**–**F**) leaves under various pulsed light intensities. The CFI curves are presented as mean values of the relative variable fluorescence (Vt). The pulsed light intensities were 200 (**A**,**D**), 1000 (**B**,**E**), and 3000 (**C**,**F**) µmol·m^−2^·s^−1^.

**Figure 2:**
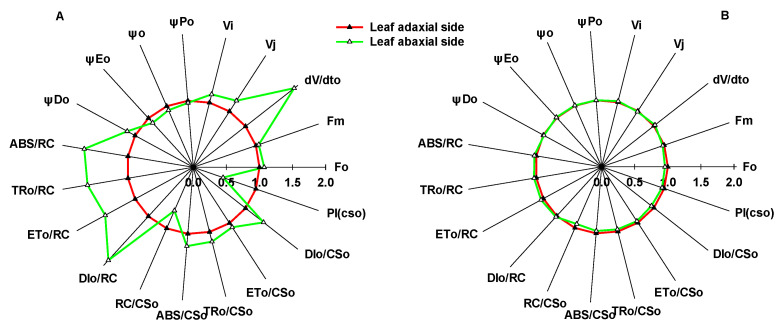
Differences in the JIP-test parameters between the adaxial and abaxial sides of sunflower (**A**) and sorghum (**B**) leaves. F_o_, minimal recorded fluorescence intensity; F_m_, maximal recorded fluorescence intensity; V_J_, relative variable fluorescence intensity at the J-step; V_I_, relative variable fluorescence intensity at the I-step; dV/dto, Q_A_ maximum reduction rate; *φ_Po_*, maximum quantum yield for primary photochemistry (at t = 0); *ψ*_o_, probability that a trapped exciton moves an electron into the electron transport chain beyond Q_A_^−^ (at t = 0); φ_Eo_, quantum yield for electron transport (at t = 0); φ_Do_, quantum yield of energy dissipation (at t = 0); ABS/RC, absorption flux per reaction center (at t = 0); TR_o_/RC, trapped energy flux per reaction center (at t = 0); ET_o_/RC, electron transport flux per reaction center (at t = 0); DI_o_/RC, dissipated energy flux per reaction center (at t = 0); ABS/CS_o_, absorption flux per cross section (at t = 0); TR_o_/CS_o_, trapped energy flux per cross section (at t = 0); ET_o_/CS_o_, electron transport flux per cross section (at t = 0); DI_o_/CS_o_, dissipated energy flux per cross section (at t = 0); RC/CS_o_, density of Q_A_-reducing PSII reaction centers; PI_CS_, performance index on cross section basis (at t = 0).

Significant differences in the CFI curves between the adaxial and abaxial sides of sunflower leaves were detected (Figure 1). As the pulsed light intensity increased, the differences in the CFI curves between the two sides of sunflower leaves gradually decreased (Figure 1A–C). In contrast, the difference between the CFI curves of the two sides of sorghum isofacial leaves was very slight under various pulsed light intensities (Figure 1D–F). There were significant differences detected in the JIP-test parameters between the two sides of sunflower leaves, and the electron transfer efficiency was significantly lower on the abaxial side than the adaxial side; no significant differences were detected between the two sides of sorghum leaves (Figure 2).

### 2.2. Effects of Enhanced Light Transmission inside Plant Leaves on the CFI Curves

**Figure 3:**
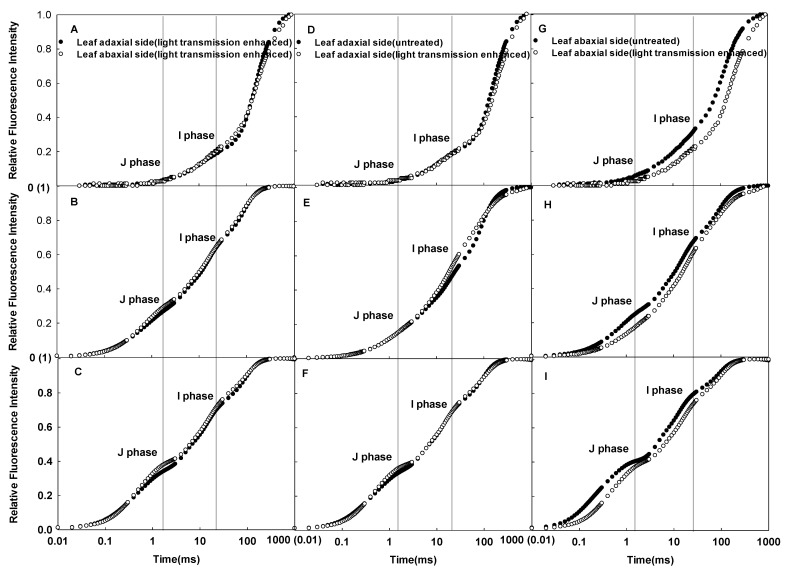
Differences in the CFI curves of the adaxial and abaxial sides of sunflower after enhancing light transmission under various pulsed light intensities (**A**–**C**); effects of enhanced light transmission on the CFI curves of the adaxial side of sunflower leaves under various pulsed light intensities (**D**–**F**); effects of enhanced light transmission on the CFI curves of the abaxial side under various pulsed light intensities (**G**–**I**). The pulsed light intensities were 200 (**A**,**D**,**G**), 1000 (**B**,**E**,**H**), and 3000 (**C**,**F**,**I**) µmol·m^−2^·s^−1^.

Although there were significant differences detected in the CFI curves between the adaxial and abaxial sides of sunflower leaves in vivo, these differences almost disappeared after enhancing the light transmission within the leaves (Figure 3A–C). Enhancing the light transmission inside the leaves had little effect on the CFI curves on the adaxial side but a great effect on the abaxial side (Figure 3D–I). Therefore, the reduced incident light scatting and refraction in spongy tissues decreased the differences in the CFI curves between the adaxial and abaxial sides.

### 2.3. Effects of Growth Irradiances on the CFI Curves

**Figure 4:**
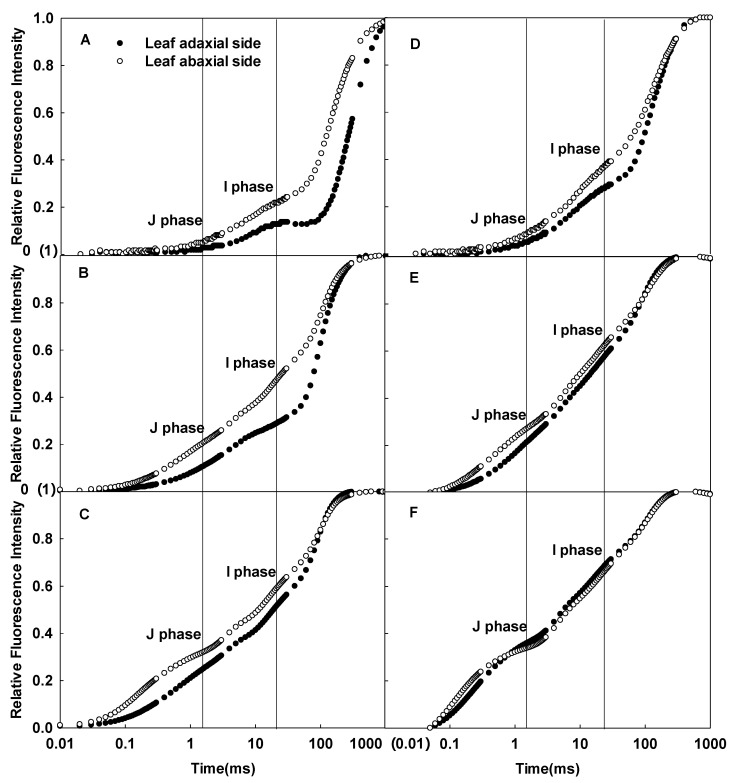
Differences in the CFI curves between the adaxial and abaxial sides of sunflower leaves grown under full sunlight (**A**–**C**) and low light (**D**–**F**). The pulsed light intensities were 200 (**A**,**D**), 1000 (**B**,**E**), and 3000 (**C**,**F**) µmol·m^−2^·s^−1^.

**Figure 5:**
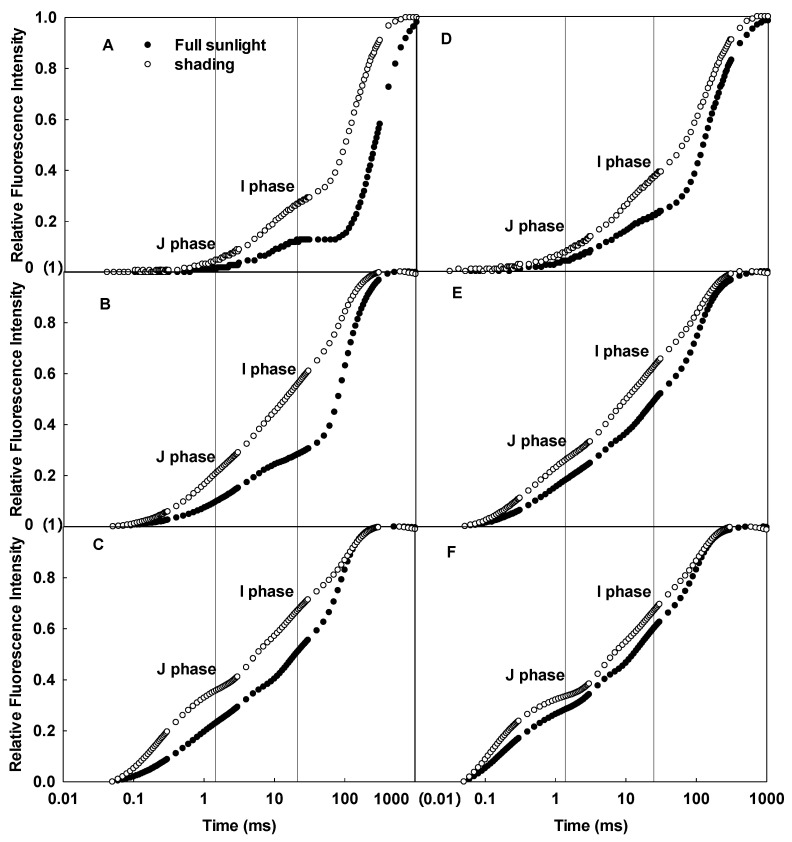
Effects of full sunlight and low light (10% full sunlight) on the CFI curves of the adaxial (**A**–**C**) and abaxial (**D**–**F**) sides of sunflower leaves. The intensities of pulsed light were 200 (**A**,**D**), 1000 (**B**,**E**), and 3000 (**C**,**F**) µmol·m^−2^·s^−1^.

The CFI curves of the adaxial and abaxial sides of sunflower leaves significantly differed when grown under full sunlight (Figure 4A–C), but the differences between the two sides decreased in the shaded group (Figure 4D–F). Compared to full sunlight, the J and I phases of the CFI curves of the adaxial and abaxial sides increased in sunflower leaves grown in low light, whether under 200, 1000, or 3000 µmol·m^−2^·s^−1^ pulsed light; the increase was greater in the adaxial side (Figure 5). Clearly, growth under low light resulted in more significant changes in the CFI curves of the adaxial side of the leaves.

### 2.4. Differences in Leaf Microstructure and Photosynthetic Rates

**Figure 6:**
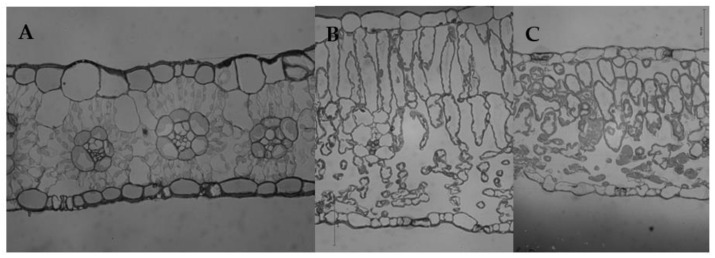
Microstructure of sorghum and sunflower leaves. (**A**) Sorghum grown under full sunlight. (**B**) Sunflower grown under full sunlight. (**C**) Sunflower grown under weak light (10% full sunlight).

No obvious differences were detected between the palisade and spongy tissues in sorghum leaves (Figure 6A). The mesophyll cells were composed of densely arranged, irregular cells. Additionally, the vascular bundle was surrounded by one layer of sheath cells, forming a “Kranz-type anatomy”. Therefore, sorghum leaves were considered to be typical isolateral leaves. In contrast, sunflower leaves had obvious palisade and spongy tissues, indicating that they were typically bifacial or heterofacial leaves. Palisade tissues, which were close to the adaxial side, consisted of 1–2 layers of dense columnar cells; spongy tissues, which were located on the abaxial side, consisted of multi-layer, loosely arranged, irregular cells (Figure 6B). Compared to the full sunlight group, the thickness of sunflower leaves grown under shade decreased. Shading resulted in significantly shortened columnar cells in palisade tissues. Moreover, morphological differences in the spongy cells between the full sunlight and shade groups also decreased (Figure 6C).

**Figure 7:**
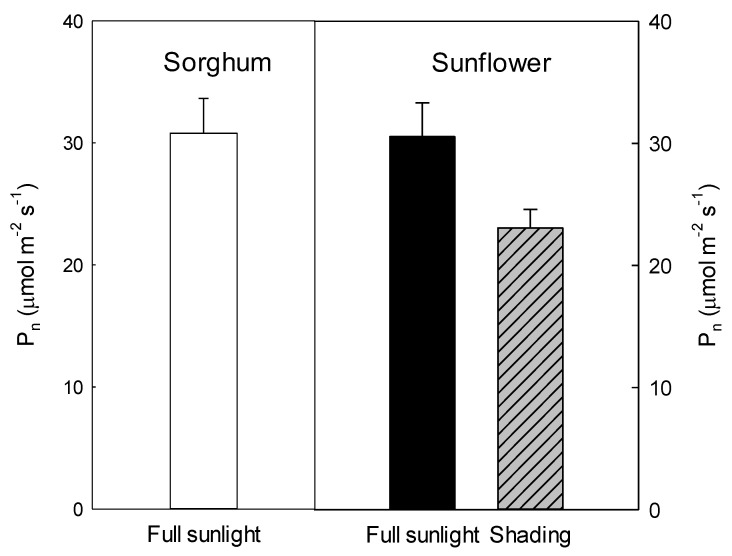
Light-saturated photosynthetic rates (P_n_) of sorghum and sunflower leaves. Plants grown under full sunlight or weak light (10% full sunlight).

To further confirm the differences between the two light treatment groups, the gas exchange was measured (Figure 6). The light-saturated photosynthetic rates of sorghum and sunflower leaves were 30 µmol·m^−2^·s^−1^ when grown under full sunlight, indicating there was little difference in the photosynthetic capacity between the two plants (Figure 7). Compared to full sunlight, shade resulted in a 20% decrease in the light-saturated photosynthetic rate (24 µmol·m^−2^·s^−1^) of sunflower leaves.

## 3. Discussion

In this study, the difference in leaf mesophyll tissue within sorghum leaves was slight, which was consistent with the slight differences detected in the CFI curves of the leaf adaxial and abaxial sides (Figure 1D–F). In contrast, there was significant palisade and spongy tissue differentiation within sunflower leaves, as well as significant differences in the CFI curves of the adaxial and abaxial sides (Figure 1A–C). This result is consistent with the fluorescence data on various other plants [24]. Chlorophyll *a* fluorescence mainly reflects the photosynthetic function of shallow cells in leaves. However, as pulsed light intensity increases, the pulsed light may penetrate through shallow leaf mesophyll tissue and release fluorescence from deeper chloroplasts [23,25,26]. In this study, to ensure that the fluorescence signal originated from shallow tissue cells, the pulsed light intensity was sequentially reduced. The difference in the CFI curves between the adaxial and abaxial sides of sunflower leaves was more pronounced under weak pulsed light. The fluorescence yields of the J and I phases were higher on the abaxial side than the adaxial side of sunflower leaves (Figure 1A–C). Thus, spongy tissues clearly had lower photosynthetic electron transfer activity per unit cross-section than palisade tissue (Figure 2). These results also suggest that the large differences in the CFI curves between the adaxial and abaxial sides of sunflower leaves were consistent with the large structural differences between palisade and spongy tissues.

We noticed that the palisade tissues of sunflower leaves under full sunlight consisted of columnar cells, while the spongy cells were round or irregular and loosely arranged (Figure 6B). This structural characteristic of spongy tissue largely led to easily scattered and refracted incident light within the leaves, which may have affected the fluorescence measurements. Previous studies have shown that the infiltrating plant leaves with water can greatly reduce the scattering and refraction caused by spongy tissue cells [27]. In this study, the difference in the CFI curves between the adaxial and abaxial sides of the leaves was greatly reduced and even disappeared after enhancing light transmission, and this reduction in the CFI curves was not dependent on pulsed light intensity (Figure 3A–C). The magnitude of change in the CFI curves of the abaxial side of sunflower leaves was greater than the adaxial side (Figure 3D–I). These results clearly indicate that the scattering and refraction of incident light due to the structural properties of spongy tissue significantly affected the determination of CFI. It is likely that the same pulsed light within the leaves may have excited the same number of chloroplasts after excluding the effects of leaf structure. Indeed, this notion is supported by the reduced CFI differences between the adaxial and abaxial sides of sunflower leaves under strong pulsed light. After excluding light refraction due to leaf structure, the difference in photosynthetic electron transfer activity of PSII between palisade and spongy tissues decreased.

The cells of sunflower leaves became smaller, and the difference between palisade and spongy tissues decreased when grown under low light (Figure 6C); the difference between the CFI curves of the leaf adaxial and abaxial sides was also significantly reduced (Figure 4). These results indicated that the growth light level affected the leaf structure and significantly changed the differences in the CFI curves of the adaxial and abaxial sides of the leaves. Among them, changes in the palisade tissues in the structure of leaves grown under low light were the most obvious when compared to sunflower leaves grown under full sunlight (Figure 6B,C). Consistently, the shape of the CFI curves of sunflower leaves grown under low light also changed significantly on the adaxial side, while changes on the abaxial side were relatively small (Figure 5). At this time, the J and I phases of the CFI curves of the adaxial side of leaves grown under low light were significantly higher than leaves grown under full sunlight; this trend was especially obvious when measured under weak pulsed light. Therefore, this indicates that the structural changes in the palisade tissue of leaves due to growth irradiance were related to changes in the CFI curves on the adaxial side, which may further affect differences in the CFI curves on both sides of the leaves.

**Figure 8:**
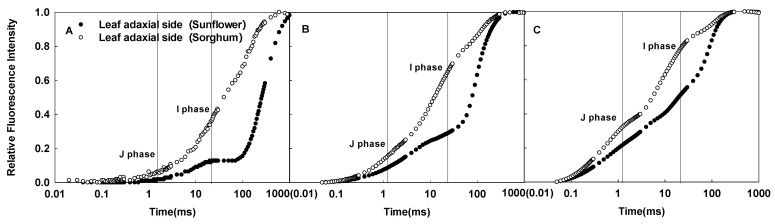
Differences in the CFI curves of the adaxial sides of sunflower and sorghum leaves under various pulsed light intensities. The CFI curves are presented as means values of the relative variable fluorescence (V_t_). The pulsed light intensities were 200 (**A**), 1000 (**B**), and 3000 (**C**) µmol·m^−2^·s^−1^.

In this study, significant differences in the CFI curves of the adaxial side of sunflower and sorghum leaves were detected (Figure 8). The JIP-test parameters indicated that sorghum leaves had a lower electron transfer efficiency than sunflower leaves (Figure 2), which is inconsistent with similar photosynthetic rates of both species. Additionally, we found that sorghum leaf mesophyll cells on the adaxial side were irregular and tightly arranged and significantly differed from columnar palisade tissue cells on the adaxial side of sunflower leaves (Figure 6). In other words, differences in the CFI curves of sunflower and sorghum leaves were consistent with their structural differences on the adaxial side. Therefore, the specific structure of sorghum leaves led to altered CFI curve shapes. Data on other plant species also support our conclusions (unpublished data), proving that the effects of leaf structure on the CFI curves are universal.

Many environmental stressors decrease the electron transport activity of PSII in vivo, including high light, salt stress, and drought [28,29,30]. However, leaf structure also changes to some extent under environmental stress, including cell miniaturization, and columnar cells are no longer evident [31]. According to our study, these structural changes likely enhance incident light refraction, which in turn enhances the J and I phases of the CFI curves, thereby decreasing photosynthetic electron transfer activity, as indicated by the JIP-test parameters. By excluding the possible effects of leaf structure on incident light refraction, changes in the PSII activity can be exactly reflected. Therefore, previous studies may have overestimated the magnitude of changes in the PSII electron transfer activity under various stress conditions to some extent. 

Thus, we suggest that large differences or variations in leaf structure may affect the shape of the CFI curves in sunflower leaves, which may further affect calculations of PSII electron transfer activity.

## 4. Materials and Methods

### 4.1. Plant Materials

The experiment was conducted at the Institute of Botany, Chinese Academy of Sciences, from May to September in 2020 and 2021. Sunflower and sorghum were planted in pots (29 cm diameter, 30 cm height). The substrate in the pots consisted of grass charcoal and soil (1:1, *v*/*v*). Seedlings were placed outdoors and divided into strong light (full sunlight) and low light (10% full sunlight obstructed by shading) groups. On sunny days, the daily maximum photosynthetic photon flux density (PPFD) was 1400–1600 μmol m^−2^ s^−1^ at midday. Normal water was provided, and fertilizer management was performed throughout the experiment to avoid nutrient and drought stress. After plants had 7–8 leaves, all measurements were conducted using just fully expanded leaves.

### 4.2. Determination of Gas Exchange

Gas exchange was measured at an irradiance of 1200 µmol·m^−2^·s^−1^ using a portable photosynthesis system (Ciras-2; PP Systems, Amesbury, MA, USA) from 08:00 to 12:00 on a sunny day. During this process, CO_2_ concentration and humidity were maintained at 380 ± 20 μmol mol^−1^ and 75% ± 5%, respectively. An ambient temperature was maintained in the leaf chamber. Five replicates from each group were used for the measurements.

### 4.3. Determination of CFI

CFI was measured using a Plant Efficiency Analyzer (PEA) (Hansatech Instruments Ltd., Norfolk, UK). Fluorescence curves were recorded during 1 s pulses of red radiation. All samples were detached and fully dark-adapted (1 h) before measurements were taken. The first reliably measured point of the CFI was at 20 µs, which was used as the minimum fluorescence (F_O_). The following data were obtained: fluorescence intensity at 2 ms (J phase (F_J_)), fluorescence intensity at 30 ms (I phase (F_I_)), and maximum fluorescence intensity (F_P_). The relative variable fluorescence was calculated as, V_t_ = (F_t_ − F_O_)/(F_P_ − F_O_) [32], where F_t_ is the measured fluorescence intensity at time t between F_O_ and F_P_. The pulsed light intensities were 200, 1000, and 3000 μmol m^−2^ s^−1^. Twenty replicates were used for each pulsed light intensity measurement. All JIP-test parameters (Table 1) were calculated following previously described methods [5].

### 4.4. Enhancement of Light Transmission within Leaves

Leaf discs were cut out of sunflower leaves and placed in a beaker with distilled water, and then pumped with a vacuum pump until the leaves were submerged in water [27]. CFI was subsequently measured using the leaf discs.

### 4.5. Determination of Leaf Structure

Leaf segments (2 × 2 mm) without major veins were cut from the basal part of the leaf lamina using a razor blade. The segments were fixed in 0.1 µL PBS solution (3% glutaraldehyde, 1% paraformaldehyde), rinsed three times with PSB solution, fixed overnight with <1% starvation acid, rinsed three times with PBS solution, dehydrated in an ethanol series, embedded in Spur resin, and cut into l µm thick sections. Then, the sections were stained with toluidine blue, observed under a light microscope (Nikon-E800), and photographed with a digital camera.

### 4.6. Statistical Analysis

Data were analyzed using one-way ANOVA and compared with the significant difference (LSD) multiple comparison test using SPSS (version 25). The least significant differences between the means were estimated at a 95% confidence level. Plots and curves were generated using the graphics software Sigmaplot v12.5.

## 5. Conclusions

Large differences or variations in leaf structure may affect the shape of the CFI curves in sunflower leaves, which may further affect calculations of JIP-test parameters.

## Figures and Tables

**Table 1:** Formulae and terms used by the JIP-test for the analysis of the fluorescence transient O-J-I-P.

Formulae and Terms	Illustrations
F_o_	Minimal recorded fluorescence intensity
F_m_	Maximal recorded fluorescence intensity
V_J_ ≡ (F_J_ − F_o_)/(F_m_ − F_o_)	Relative variable fluorescence intensity at the J-step
V_I_ ≡ (F_I_ − F_o_)/(F_m_ − F_o_)	Relative variable fluorescence intensity at the I-step
dV/dto	Q_A_ maximum reduction rate
*φ_Po_* ≡ TR_o_/ABS = [1 − (F_o_/F_m_)]	Maximum quantum yield for primary photochemistry (at t = 0)
*ψ*_o_ ≡ ET_o_/TR_o_ = (1 − V_J_)	Probability that a trapped exciton moves an electron into the electron transport chain beyond Q_A_^−^ (at t = 0)
φ_Eo_ ≡ ET_o_/ABS = [1 − (F_o_/F_m_)]·*ψ*_o_	Quantum yield for electron transport (at t = 0)
φ_Do_ ≡ 1 − *φ_Po_* = (F_o_/F_m_)	Quantum yield (at t = 0) of energy dissipation
ABS/RC = M_0_ (1/V_J_) (1/*φ_Po_*)	Absorption flux per RC
TR_o_/RC = M_0_ (1/V_J_)	Trapped energy flux per RC (at t = 0)
ET_o_/RC = M_0_ (1/V_J_) *ψ*_o_	Electron transport flux per RC (at t = 0)
DI_o_/RC = (ABS/RC) − (TR_o_/RC)	Dissipated energy flux per RC (at t = 0)
ABS/CS_o_ ≈ F_o_	Absorption flux per CS (at t = 0)
TR_o_/CS_o_ = *φ_Po_*·(ABS/CS_o_)	Trapped energy flux per CS (at t = 0)
ET_o_/CS_o_ = *φ*_Eo_·(ABS/CS_o_)	Electron transport flux per CS (at t = 0)
DI_o_/CS_o_ = (ABS/CS_o_) − (TR_o_/CS_o_)	Dissipated energy flux per CS (at t = 0)
Density of reaction centers	
RC/CS_o_ = *φ_Po_*·(V_J_/Mo)·(ABS/CS_o_)	Density of RCs (Q_A_-reducing PSII reaction centers)
PI_CS_ ≡ (RC/CS_o_) [*φ_Po_*/(1 − *φ_Po_*)] [*ψ*_o_/(1 − *ψ*_o_)]	Performance index on cross section basis (at t = 0)

## Data Availability

Not applicable.
